# Human Immunodeficiency Virus Awareness Among First-Year Medical Students at the Puerto Rican School of Medicine: A Cross-Sectional Study

**DOI:** 10.7759/cureus.107601

**Published:** 2026-04-23

**Authors:** Juan Salichs, Sofia Rey-Garaycoa, Linda Pérez-Laras, Guillermo Martinez Hernandez, Yarelis Martinez Rodriguez, Peter Barbosa

**Affiliations:** 1 Biomedical Sciences, San Juan Bautista School of Medicine, Caguas, PRI; 2 Puerto Rico Health Justice Center, San Juan Bautista School of Medicine, Caguas, USA; 3 Basic Sciences, Ponce Health Sciences University - St. Louis Campus, St. Louis, USA

**Keywords:** first-year medical student, hiv, hiv prevention, medical education, public health

## Abstract

Introduction

Human immunodeficiency virus (HIV) remains a pressing global public health challenge, particularly in Puerto Rico, where prevalence rates outpace most U.S. jurisdictions. Despite advances in antiretroviral therapy (ART) and the availability of biomedical prevention strategies, such as pre-exposure prophylaxis (PrEP) and the Undetectable = Untransmittable (U=U) campaign, gaps in local knowledge persist among future healthcare providers.

Methods

A cross-sectional study was conducted at San Juan Bautista School of Medicine, Caguas, Puerto Rico, among first-year medical students. Participants completed a three-part anonymous online survey via REDCap, capturing demographics, general HIV knowledge using the validated HIV-KQ-18, and awareness of recent prevention concepts. Descriptive statistics were used to summarize sociodemographic variables and HIV knowledge. Sociodemographic predictors of HIV knowledge were selected using the Least Absolute Shrinkage and Selection Operator (LASSO) regression.

Results

Eighty-two students participated, with a mean age of 23.4 years and 59.8% female. The mean HIV-KQ-18 score was 14.5 out of 18, reflecting moderate knowledge. Key deficits included awareness of PrEP (40.2% answered correctly) and understanding of the U=U campaign (53.7% answered correctly). Sociodemographic predictors of HIV knowledge included political views, gender, highest education level before beginning the MD program, location raised, religious beliefs, family household income, and living status.

Conclusions

Significant gaps in HIV prevention and transmission knowledge were identified among first-year medical students in Puerto Rico, underscoring a need for enhanced, culturally responsive educational initiatives focused on biomedical prevention and combating misinformation. These findings should inform targeted curricular reforms and public health outreach in similar high-prevalence regions.

## Introduction

Infection with the human immunodeficiency virus (HIV) continues to be a major global public health concern, with approximately 42.3 million deaths attributed since its discovery [1]. Despite advancements in medical care, including the widespread availability of antiretroviral therapy (ART), HIV prevention remains a crucial element in combating the epidemic. Pre-exposure prophylaxis (PrEP), post-exposure prophylaxis (PEP), and the Undetectable = Untransmittable (U=U) campaign represent some of the most significant strides in prevention, providing effective strategies for reducing transmission rates, particularly in high-risk populations [2,3]. However, despite these advancements, Puerto Rico experiences one of the highest HIV prevalence rates in the United States, with approximately 1.1% of the adult population living with HIV. Puerto Rico also faces significant gaps in PrEP use compared to the mainland U.S. [4].

In the context of medical education, understanding and applying HIV prevention strategies are essential for future healthcare providers. First-year medical students, poised to enter the medical workforce, represent an ideal cohort for assessing baseline knowledge and readiness to engage in HIV prevention efforts. This study evaluates HIV knowledge among first-year medical students at San Juan Bautista School of Medicine in Caguas, Puerto Rico, focusing on their understanding of prevention methods and transmission. By identifying demographic variables correlated with knowledge gaps, this study aims to inform educational and outreach efforts designed to enhance HIV understanding and reduce misinformation among future healthcare professionals.

The study underscores the need for more comprehensive HIV education within undergraduate medical curricula, particularly in prevention, transmission, and societal impacts. With Puerto Rico's unique epidemiological context - where HIV prevalence is high and PrEP utilization remains low - the findings from this study may serve as a critical baseline for enhancing public health and educational strategies aimed at reducing HIV transmission.

## Materials and methods

A cross-sectional study was designed to assess HIV-related knowledge and awareness among first-year medical students at San Juan Bautista School of Medicine (SJBSM), Caguas, Puerto Rico. Medical students from two first-year cohorts, including the Fall 2024 and Fall 2025 entering classes, completed a three-part survey. Data were collected in November 2024 and August 2025 via a self-administered, web-based survey hosted on the REDCap platform [5,6]. All participation was voluntary, and no incentives were provided. The survey was distributed to first-year medical students prior to their infectious disease courses. All data were collected anonymously to protect participants’ confidentiality. Ethical approval was obtained from the Institutional Review Board of San Juan Bautista School of Medicine (IRB: EMSJBIRB-13-2024). Inclusion criteria were active, enrolled medical students in their first academic year and access to either a smartphone or a tablet. Exclusion criteria included incomplete surveys or missing primary outcome measures. No incentives were provided.

This study evaluated the distribution of HIV knowledge scores and knowledge of recent HIV preventive measures and identified potential predictors of knowledge based on socioeconomic and demographic characteristics. The survey was divided into three main sections: (1) demographic information, (2) a previously published and validated HIV Knowledge Questionnaire (HIV-KQ-18) [7], and (3) a seven-question, newly developed instrument about contemporary HIV preventive measures.

The entire survey is presented in Appendix 1. Section 2 of the survey was validated as previously described [7]; the survey instrument is being piloted for Sections 1 and 3.

Variables

Section 1

Sociodemographic variables included age (categorized as 20-22, 23-25, 26-28, and ≥29 years), gender identity, sexual orientation, race/ethnicity, prior educational attainment, undergraduate major, household income, relationship status, number of sexual partners in the past year, primary language, religious affiliation, place of birth and upbringing, living situation, political views, family composition, household size during childhood, and pre-medical work experiences.

Section 2

General HIV knowledge was assessed using the validated HIV-KQ-18, yielding a total score ranging from 0 to 18, with higher scores indicating greater knowledge [7]. Each item had three response options: “True,” “False,” or “I don’t know.” Topics covered included transmission myths, preventive measures, and medical misconceptions. Correct answers were scored as 1; incorrect answers and “I don’t know” were scored as 0. Combined scores were categorized into three levels: low (0-10), moderate (11-15), and high (16-18).

Section 3

The final section of the survey assessed knowledge of current HIV prevention measures. Awareness of recent HIV prevention measures was evaluated using items assessing understanding of U=U, knowledge of PrEP and PEP, and risk-reduction strategies.

Statistical analysis

Descriptive statistics were used to summarize sociodemographic variables. Frequency and prevalence were reported for categorical data. No formal sample-size calculation was performed because the study aimed to survey the first-year MD student population. All completed questionnaires were included in the final analysis.

The Least Absolute Shrinkage and Selection Operator (LASSO) regression was applied as a variable selection method to screen the complete set of sociodemographic variables [8]. Ten-fold cross-validation was used to determine the optimal penalty parameter (λ) that minimized the cross-validated prediction error. Analyses were conducted using Stata Statistical Software, Release 19 (2023; StataCorp LLC, College Station, TX, USA). Significance was set at p < 0.05, and 95% confidence intervals were reported where applicable.

## Results

The total number of participants was 82. The mean age of the participants was 23.4 years. Participants were 59.8% female, and most were raised in Puerto Rico (67.1%). Additional participant demographics are shown in Table 1.

**Table 1:** Survey Section 1 - Participant Demographics

Characteristics	N = 82 (%)
Age	
Mean	23.4 years (SD ±3.12)
24 and younger	63 (76.8)
25 and older	19 (23.2)
Gender	
Female	49 (59.8)
Male	32 (39.0)
Non-binary	1 (1.22)
Sexual orientation	
Heterosexual	69 (84.2)
Bisexual	9 (11.0)
Homosexual	2 (2.4)
Asexual	2 (2.4)
Highest education level before beginning the MD program	
Bachelor’s Degree	70 (85.4)
Master’s Degree	10 (12.2)
Doctoral or Professional Degree	2 (2.4)
Major before beginning the MD program	
Biological/Biomedical Sciences	70 (85.4)
Public Health	3 (3.6)
Chemistry	2 (2.4)
Physiology/Kinesiology	2 (2.4)
Liberal Arts	1 (1.2)
Engineering	1 (1.2)
Neuroscience	1 (1.2)
Microbiology	1 (1.2)
Psychology	1 (1.2)
Location major before beginning the MD program	
Puerto Rico	50 (61.0)
Mainland United States	32 (39.0)
Family household income growing up with the family	
Less than $25,000	10 (12.2)
$25,000 - $49,999	12 (14.6)
$50,000 - $74,999	21 (25.6)
$75,000 - $99,999	12 (14.6)
$100,000 - $149,999	9 (11)
$150,000 or more	18 (22)
Current relationship status	
Married	9 (11)
Committed Relationship	27 (32.9)
Single	46 (56.1)
Sexual partners in the past (n = 81)	
None	15 (18.3)
1-2	56 (68.3)
3-5	7 (8.5)
5+	3 (3.7)
Preferred language	
Spanish	48 (58.5)
English	34 (41.5)
Religious belief	
Catholicism	42 (51.2)
Protestant/Evangelical	20 (24.4)
Islam	3 (3.7)
Agnostic	6 (7.3)
Spiritual non-religious	5 (6.1)
Atheism	5 (6.1)
The Church of Jesus Christ of Latter-Day Saints	1 (1.2)
Place of birth	
Puerto Rico	54 (65.9)
Mainland US	24 (29.3)
Other	4 (4.9)
Location raised	
Puerto Rico	55 (67.1)
Mainland US	25 (30.5)
Other	2 (2.4)
Living status	
Renter	43 (52.4)
Living with parents	35 (42.7)
Homeowner	3 (3.7)
Relative’s home	1 (1.2)
Political views	
Liberal	42 (51.2)
Moderate	29 (35.4)
Conservative	11 (13.4)
Raised by	
Two parents: Mother and Father	72 (87.8)
One parent: Mother	10 (12.2)
Household (including MD student)	
Small household (1-2 persons)	19 (23.2)
Medium household (3-4 persons)	54 (65.9)
Large household (5 or more persons)	9 (11)
Prior work experience	
Research assistant - clinical research	34 (41.5)
Research assistant - laboratory research	43 (52.4)
Medical assistant	23 (28.1)
Medical scribe	13 (15.9)
Medical sales	1 (1.2)
Shadowing	75 (91.5)
Tutoring/Teaching assistant	37 (45.1)
Interpreter	1 (1.2)
Volunteer work	1 (1.2)
Construction	1 (1.2)
Nursing assistant	1 (1.2)
Pathologist (PT technician)	1 (1.2)
Public health	1 (1.2)

**Figure 1:**
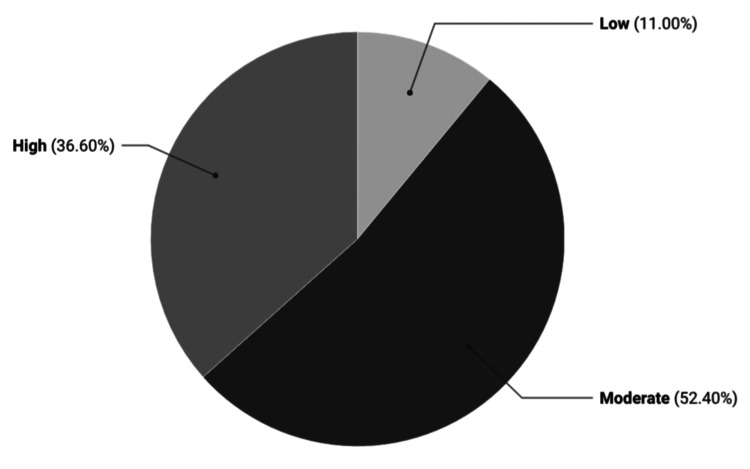
Human Immunodeficiency Virus Knowledge Levels Based on HIV-KQ-18

Responses to the validated HIV-KQ-18 are detailed in Table 2. The mean HIV-KQ-18 score was 14.5 (SD 2.8; range 0-18). Using categorization thresholds, 11% of participants demonstrated poor knowledge, 52.4% moderate knowledge, and 36.6% high knowledge. The distribution of total scores is presented in Figure 1.

**Table 2:** Survey Section 2 - HIV Knowledge Questionnaire Responses

Survey question	True, n (%)	False, n (%)	I don’t know, n (%)	Correct answer
Coughing and sneezing DO NOT spread HIV.	57 (69.5)	15 (18.3)	10 (12.2)	TRUE
A person can get HIV by sharing a glass of water with someone who has HIV.	15 (18.3)	61 (74.4)	6 (7.3)	FALSE
Pulling out the penis before a man climaxes/cums keeps women from getting HIV during sex.	0	79 (97.5)	2 (2.5)	FALSE
A woman can get HIV if she has anal sex with a man.	73 (89.0)	1 (1.2)	8 (9.8)	TRUE
Showering, or washing one's genitals/private parts, after sex keeps a person from getting HIV.	4 (4.9)	71 (86.6)	7 (8.5)	FALSE
All pregnant women infected with HIV will have babies born with AIDS	10 (12.2)	58 (70.7)	14 (17.1)	FALSE
People who have been infected with HIV quickly show serious signs of being infected.	1 (1.22)	75 (91.5)	6 (7.3)	FALSE
There is a vaccine that can stop adults from getting HIV.	15 (18.3)	54 (65.9)	13 (15.9)	FALSE
People are likely to get HIV by deep kissing, putting their tongue in their partner's mouth, if their partner has HIV.	20 (24.4)	52 (63.4)	10 (12.2)	FALSE
A woman cannot get HIV if she has sex during her period.	1 (1.2)	77 (93.9)	4 (4.9)	FALSE
There is a female condom that can help decrease a woman's chance of getting HIV.	71 (86.6)	2 (2.4)	9 (11)	TRUE
A natural skin condom works better against HIV than does a latex condom.	1 (1.2)	51 (62.2)	30 (36.6)	FALSE
A person will NOT get HIV if she or he is taking antibiotics.	0	74 (90.2)	8 (9.8)	FALSE
Having sex with more than one partner can increase a person's chance of being infected with HIV.	80 (97.6)	2 (2.4)	0	TRUE
Taking a test for HIV one week after having sex will tell a person if she or he has HIV.	8 (9.8)	51 (62.2)	23 (28.1)	FALSE
A person can get HIV by sitting in a hot tub or a swimming pool with a person who has HIV.	2 (2.4)	70 (85.4)	10 (12.2)	FALSE
A person can get HIV from oral sex.	64 (78.1)	8 (9.8)	10 (12.2)	TRUE
Using Vaseline or baby oil with condoms lowers the chances of getting HIV.	3 (3.7)	71 (86.6)	8 (9.8)	FALSE

Responses to the third survey section, which assesses knowledge of current HIV prevention measures, are detailed in Table 3. Over half of respondents (53.7%) correctly identified the meaning of U=U. Thirty-three (40.2%) accurately reported that PrEP reduces the risk of contracting HIV in high-risk individuals.

**Table 3:** Survey Section 3 - Survey Responses to HIV Prevention Measures Knowledge

Self-report HIV prevention measures knowledge	n (%)	Correct answer
Q.20) Which of the following methods can effectively reduce the risk of HIV transmission? (Select all that apply)		
Using condoms during sex (n = 82)	79 (96.3)	TRUE
Taking medication (n = 82)	42 (51.2)	TRUE
Abstinence from sexual activity (n=82)	79 (96.3)	TRUE
Using oral contraceptives (n = 82)	15 (18.3)	FALSE
Avoid sharing needles (n = 82)	77 (93.9)	TRUE
Taking the HIV vaccine (n = 82)	39 (47.6)	FALSE
Not sure (n = 82)	2 (2.44)	N/A
Q.21) What does U=U mean?		
Undetectable = Untransmittable	44 (53.7)	TRUE
Untraceable = Unaffected	7 (8.5)	FALSE
Unidentifiable = U don't have it	1 (1.2)	FALSE
Not sure	30 (36.6)	FALSE
Q.22) What is pre-exposure prophylaxis (PrEP) used for?		
To treat active HIV infection	4 (4.9)	FALSE
To reduce the risk of contracting HIV in individuals at high risk	33 (40.2)	TRUE
Preventing the transmission of HIV after potential exposure	12 (14.6)	FALSE
To cure individuals with AIDS	0	FALSE
To prevent bacterial sexually transmitted infections (STIs)	1 (1.2)	FALSE
To prevent viral sexually transmitted infections (STIs)	1 (1.2)	FALSE
Not sure	31 (37.8)	N/A
Q.23) Is a negative HIV test result required to receive PrEP?		
True	17 (20.7)	TRUE
False	23 (28.1)	FALSE
Not sure	43 (52.4)	N/A
Q.24) In your understanding, what is the primary purpose of post-exposure prophylaxis (PEP)?		
Preventing the transmission of HIV after potential exposure	17 (20.7)	TRUE
To reduce the risk of contracting HIV in individuals at high risk	26 (31.7)	FALSE
Treating an existing HIV infection	4 (4.9)	FALSE
Preventing sexually transmitted infections in general	3 (3.7)	FALSE
Not sure	32 (39.0)	N/A
Q.25) A person with HIV who has an undetectable viral load cannot transmit the virus to others through sexual contact.		
True	33 (40.2)	TRUE
False	36 (43.9)	FALSE
Not sure	13 (15.9)	N/A

Sociodemographic predictors of HIV knowledge (HIV-KQ-18) were selected using the LASSO regression. Using 10-fold cross-validation, the optimal penalty parameter was λ = 0.153, which minimized the mean cross-validated error. Eleven sociodemographic variables were shrunk to zero, indicating limited contribution to predicting HIV knowledge. The seven variables retained with non-zero coefficients at the optimal λ included political views, gender, highest level of education before beginning the MD program, location raised, religious beliefs, family household income, and living status. The coefficients for all candidate predictors at the optimal λ are presented in Table 4. The non-binary category was removed from the LASSO analysis.

**Table 4:** LASSO Regression Analysis for Predictors of HIV Knowledge Score LASSO: Least Absolute Shrinkage and Selection Operator

Sociodemographic	Coefficient at λ_min	Selected (non-zero)
Political views	0.98	Yes
Gender	0.68	Yes
Highest education level before beginning the MD program	0.42	Yes
Location raised	0.42	Yes
Religious belief	0.35	Yes
Family household income	0.32	Yes
Living status	0.27	Yes
Age	-	No
Sexual orientation	-	No
Major before beginning the MD program	-	No
Location major before beginning the MD program	-	No
Current relationship status	-	No
Sexual partners in the past	-	No
Preferred language	-	No
Place of birth	-	No
Number of parents when raised	-	No
Number in the households (including MD students)	-	No
Prior work experience	-	No

## Discussion

The mean HIV-KQ-18 score of 14.5 (SD ±2.8) out of 18 suggests that most students were generally familiar with HIV transmission and prevention; yet over 10% demonstrated poor knowledge, and only about one-third reached high proficiency. Misconceptions about vaccines, casual contact, and diagnostic testing point to persistent foundational gaps despite biomedical-focused undergraduate training. Knowledge of biomedical prevention was particularly limited - fewer than half accurately understood PrEP’s purpose, U=U, or the prerequisites for prophylaxis.

Sociodemographic patterns indicate that political views, gender, education, upbringing, religion, income, and living status influence HIV knowledge more than prior clinical exposure or scientific coursework. These findings align with prior U.S. and Latin American studies documenting uneven understanding among health professions students, particularly regarding PrEP, PEP, and contemporary prevention paradigms [9-11].

CDC AHEAD data show Puerto Rico’s estimated HIV incidence rate in 2022 was higher than the U.S. average, around the high single digits per 100,000 population [12,13]. The Puerto Rico HIV/AIDS Surveillance Summary (Departamento de Salud) reports decades of cumulative burden: nearly 40,000 AIDS cases reported by early 2025, reflecting long-standing transmission and mortality [14,15]. Analyses of AIDSVu data classify Puerto Rico, along with Mississippi, South Carolina, Alabama, and West Virginia, as having among the lowest PrEP-to-need ratios (PNRs) in the U.S., meaning relatively few PrEP users relative to the level of new HIV diagnoses [16,17].

In Puerto Rico - where HIV prevalence is among the highest in U.S. jurisdictions and PrEP use remains extremely low - students’ uncertainty about PrEP and U=U has critical implications [11,18]. Future physicians unprepared to discuss or prescribe prevention tools may miss opportunities to screen patients or advocate for broader access. This gap reflects a generational shift: many students, born after the early HIV crisis, may perceive HIV as a manageable disease rather than a defining public health emergency, risking historical amnesia about the human, social, and ethical dimensions of the epidemic.

Research across various global contexts consistently indicates that, while medical students often possess a high level of general knowledge regarding HIV/AIDS, significant gaps and misconceptions persist, particularly concerning specific transmission routes and preventive measures. For instance, studies in Israel and Jordan highlighted inadequacies in understanding HIV transmission via breastfeeding and the prevention of vertical transmission [19,20]. Similarly, clinical students in Croatia and Vietnam demonstrated gaps in accurately estimating transmission risks following needle-stick injuries and unprotected intercourse [21,22]. These knowledge deficits are frequently coupled with stigmatizing attitudes; many students associate HIV with shame and fear or support discriminatory practices such as the mandatory disclosure of a patient's HIV status. The findings from these international medical school settings are analogous to the present study, in which most students demonstrated moderate knowledge of HIV.

Recent research highlights a significant discrepancy between high general awareness and low technical knowledge of HIV PrEP among healthcare professional students in the U.S. [23,24]. While pharmacy students demonstrate superior knowledge regarding prescribing guidelines and pharmacological details, medical students report greater comfort with clinical implementation and a higher willingness to refer candidates to other providers. Despite these disciplinary strengths, critical misconceptions remain across the student population; for instance, approximately one-third of students fail to identify HIV-negative status as a prerequisite for PrEP, and over 60% incorrectly believe that individuals with an undetectable viral load can still transmit the virus sexually [23]. Knowledge levels are notably higher among students in later stages of their training and those identifying as gay or lesbian, suggesting that current standardized curricula may be insufficient in addressing HIV prevention [23]. Collectively, these findings underscore an urgent need to enhance medical and pharmacy education to bridge knowledge gaps and ensure that future practitioners can effectively contribute to national goals for ending the HIV epidemic. These U.S.-based findings, along with those presented in this study in Puerto Rico, support the call for curricular reform.

Integrating evidence-based HIV education into medical curricula should be extended to encompass other social science domains, such as prevention science and sociocultural determinants. Targeted modules on PrEP, PEP, U=U, and patient communication - paired with reflection on personal values and biases - may enhance both cultural competence and clinical readiness. Medical schools can play a pivotal role by linking students with community health centers and public health agencies to provide early exposure to PrEP prescribing pathways and multidisciplinary prevention teams. Training that normalizes sexual health discussions and equips students to explain U=U clearly could strengthen prevention efforts amid limited prescribing capacity.

This study has several important limitations that should be considered when interpreting the findings. First, its single-site, cross-sectional design and modest sample of first-year medical students from two entering cohorts at a single Puerto Rican medical school limit generalizability to other institutions, health professions, and more advanced trainees. Second, the use of a self-administered, anonymous online survey introduces potential selection bias, as students with greater interest in HIV or public health may have been more likely to participate, and social desirability bias may have influenced responses regarding sensitive topics such as sexual behavior and prevention practices. Third, although the HIV-KQ-18 is a validated instrument, the newly developed items assessing PrEP, PEP, and U=U knowledge have not yet undergone extensive psychometric validation in this population, which may affect the precision with which these constructs were measured. Fourth, the absence of a formal sample-size calculation and the use of LASSO regression with a relatively small number of observations may have led to overfitting or unstable predictor estimates, and causality cannot be inferred from the identified associations. Finally, knowledge was assessed prior to formal infectious disease instruction and without longitudinal follow-up, preventing evaluation of how curricular exposure or clinical experiences may change HIV-related knowledge and awareness over time.

## Conclusions

This study provides one of the first systematic assessments of HIV knowledge - including PrEP, PEP, and U=U - among Puerto Rican medical students, using a validated instrument prior to formal instruction. Although limited by a single-site, self-reported, cross-sectional design, the results highlight actionable educational gaps. Future multi-institutional and longitudinal research should test structured curricula and simulated counseling encounters to identify strategies that best prepare future clinicians to combat HIV in high-prevalence, low-PrEP-use settings.

## Appendices

Appendix 1: HIV awareness survey

Section 1: Demographics

What is your age? _______

What gender do you identify as?

Male

Female

Non-binary

Other (please specify): ___________

What is your sexual orientation?

Heterosexual (straight)

Homosexual (gay)

Homosexual (lesbian)

Bisexual

Other (please specify): ___________

What is your race?

White

Black or African American

Asian or Asian American

Native Hawaiian or Other Pacific Islander

American Indian or Alaska Native

Other (please specify): ___________

What is your ethnicity?

Hispanic/Latino

Puerto Rican

Cuban

Mexican

Not Hispanic/Latino

What is your highest level of education?

Bachelor’s Degree

Master’s Degree

Doctoral or Professional Degree

Previous Medical School Registration

Other (please specify): ___________

What was your major in your highest level of education?

Biological/Biomedical Sciences

Liberal Arts

Psychology

Business/Finance

Engineering

Public Health

Other (please specify): ___________ 

Where did you complete your bachelor’s degree?

Puerto Rico

United States

State: ______

Other (please specify): ________

What was your family's household income growing up?

Less than $25,000

$25,000 - $49,999

$50,000 - $74,999

$75,000 - $99,999

$100,000 - $149,999

$150,000 or more

What is your current relationship status?

Single

In a committed relationship

Married

Divorced

Widowed

How many sexual partners have you had in the past year?

None

1-2

3-5

5 +

Prefer not to say

Other: ___________

What is your preferred language?

English

Spanish

Other (please specify): ___________

Do you identify with any of the following religious groups?

Catholicism

Christianity (Protestant/Evangelical)

Judaism

Islam

Buddhism

Agnostic

Atheism

Spiritual non-religious

Other (please specify): ___________

What is your place of birth?

Puerto Rico

Mainland U.S.

What state? ___________

Other (please specify): ___________

Where were you raised?

Puerto Rico

Mainland U.S.

What state? ___________

Other (please specify): ___________

Which of the following best describes your living status?

Homeowner

Renter

Living with Parents

Other (please specify): ___________

How would you describe your political view?

Liberal

Moderate

Conservative

Other (please specify): ___________

Who were you raised by?

Two parents: Mother and Father

Two parents: Mother and Mother

Two parents: Father and Father

One parent: Mother

One parent: Father

Relative (specify): ___________

Other (specify): ___________

How many people, including yourself, lived in your household growing up?

1

2

3

4

5

6

7

8+

What prior experience(s) did you acquire before medical school? CHOOSE ALL THAT APPLY

Research Assistant (clinical research)

Research Assistant (laboratory research)

Medical Assistant

Medical Scribe

Medical Sales

Shadowing

Tutoring/Teaching Assistant

Other (please specify): ___________

Section 2: HIV Knowledge Questionnaire (HIV-KQ-18)

Coughing and sneezing DO NOT spread HIV. (T)

True

False

I don’t know

A person can get HIV by sharing a glass of water with someone who has HIV. (F)

True

False

I don’t know

Pulling out the penis before a man climaxes/cums keeps a woman from getting HIV during sex. (F)

True

False

I don’t know

A woman can get HIV if she has anal sex with a man. (T)

True

False

I don’t know

Showering, or washing one’s genitals/private parts, after sex keeps a person from getting HIV. (F)

True

False

I don’t know

All pregnant women infected with HIV will have babies born with AIDS. (F)

True

False

I don’t know

People who have been infected with HIV quickly show serious signs of being infected. (F)

True

False

I don’t know

There is a vaccine that can stop adults from getting HIV. (F)

True

False

I don’t know

People are likely to get HIV by deep kissing, putting their tongue in their partner’s mouth, if their partner has HIV. (F)

True

False

I don’t know

A woman cannot get HIV if she has sex during her period. (F)

True

False

I don’t know

There is a female condom that can help decrease a woman’s chance of getting HIV. (T)

True

False

I don’t know

Using a condom during sex prevents a person from getting HIV. (T)

True

False

I don’t know

A person will NOT get HIV if she/he is taking antibiotics. (F)

True

False

I don’t know

Having sex with more than one partner can increase a person’s chance of being infected. (T)

True

False

I don’t know

Taking a test for HIV one week after having sex will tell a person if she/he has HIV. (F)

True

False

I don’t know

A person can get HIV by sitting in a hot tub or a swimming pool with a person who has HIV. (F)

True

False

I don’t know

A person can get HIV from oral sex. (T)

True

False

I don’t know

Using Vaseline or baby oil with condoms lowers the chance of getting HIV. (F)

True

False

I don’t know

Section 3: Knowledge of Current HIV Prevention Measures

Do you know anyone who is living with HIV?

Yes

No

Which of the following methods can effectively reduce the risk of HIV transmission? (Select all that apply) Answer: ABCE

Using condoms during sex

Taking medication

Abstinence from sexual activity

Using oral contraceptives

Avoiding sharing needles

Taking the HIV vaccine

I’m not sure

What does U=U mean? Answer A

Undetectable = Untransmittable

Untraceable = Unaffected

Unidentifiable = U dont have it

I’m not sure

What is PrEP used for?: Answer B

To treat active HIV infection

To reduce the risk of contracting HIV in individuals at high risk

Preventing the transmission of HIV after potential exposure

To cure individuals with AIDS

To prevent bacterial sexually transmitted infections (STIs)

To prevent viral sexually transmitted infections (STIs)

I’m not sure 

Is a negative HIV test result required to receive PrEP? (T)

True

False

I'm not sure 

In your understanding, what is the primary purpose of PEP? Answer: A

Preventing the transmission of HIV after potential exposure

To reduce the risk of contracting HIV in individuals at high risk

Treating an existing HIV infection

Preventing sexually transmitted infections in general

I am not sure

A person with HIV who has an undetectable viral load cannot transmit the virus to others through sexual contact. Answer: A

True

False

I’m not sure

## References

[1] (2026). HIV and AIDS. https://www.who.int/news-room/fact-sheets/detail/hiv-aids.

[2] 2] “US (2026). Office of Infectious Disease and HIV/AIDS Policy (OIDP). https://www.hhs.gov/oidp/index.html.

[3] (2026). PrEP, PEP, and beyond: choosing the right HIV prevention option for your life. https://aids.org/prep-pep-and-beyond-choosing-the-right-hiv-prevention-option/.

[4] (2026). Estimated HIV incidence and prevalence. https://www.cdc.gov/hiv-data/nhss/estimated-hiv-incidence-and-prevalence.html.

[5] Harris PA, Taylor R, Thielke R, Payne J, Gonzalez N, Conde JG (2009). Research electronic data capture (REDCap) - a metadata-driven methodology and workflow process for providing translational research informatics support. J Biomed Inform.

[6] Harris PA, Taylor R, Minor BL (2019). The REDCap consortium: building an international community of software platform partners. J Biomed Inform.

[7] Carey MP, Morrison-Beedy D, Johnson BT (1997). The HIV-knowledge questionnaire: development and evaluation of a reliable, valid, and practical self-administered questionnaire. AIDS Behav.

[8] Kukreja SL, Löfberg J, Brenner MJ (2006). A least absolute shrinkage and selection operator (LASSO) for nonlinear system identification. IFAC Proc.

[9] Vega-Ramirez H, Torres TS, Guillen-Diaz C (2022). Awareness, knowledge, and attitudes related to HIV pre-exposure prophylaxis and other prevention strategies among physicians from Brazil and Mexico: cross-sectional web-based survey. BMC Health Serv Res.

[10] Lima AL, Sesnik HH, Lima LV (2024). Factors associated with university students' knowledge about HIV and preand post-exposure prophylaxis. Rev Bras Enferm.

[11] Herbst JH, Kay LS, Passin WF, Lyles CM, Crepaz N, Marín BV (2007). A systematic review and meta-analysis of behavioral interventions to reduce HIV risk behaviors of Hispanics in the United States and Puerto Rico. AIDS Behav.

[12] (2026). America's HIV epidemic analysis dashboard. https://ahead.hiv.gov/puerto_rico/.

[13] (2026). HIV diagnoses, deaths, and prevalence: 2025 update. https://www.cdc.gov/hiv-data/nhss/hiv-diagnoses-deaths-and-prevalence-2025.html.

[14] (2026). Puerto Rico HIV/AIDS surveillance summary. https://www.estadisticas.pr.gov/en-us/productos/puerto-rico-hiv-aids-surveillance-summary.

[15] (2026). Puerto Rico AIDS surveillance summary: cumulative AIDS cases diagnosed as of March 31, 2025. https://www.scribd.com/document/906644048/Puerto-Rico-AIDS-Surveillance-Summary-Marzo-2025.

[16] (2026). Disparities in HIV prevention: new data reveal racial and regional gaps in PrEP usage. https://www.contagionlive.com/view/disparities-in-hiv-prevention-new-data-reveal-racial-and-regional-gaps-in-prep-usage.

[17] (2026). AIDSVu releases new data highlighting ongoing inequities in PrEP use among Black and Hispanic people and across regions of the country. https://aidsvu.org/news-updates/news-updates-aidsvu-releases-new-data-highlighting-ongoing-inequities-in-prep-use-among-black-and-hispanic-people-and-across-regions-of-the-county/.

[18] Murphy L, Bowra A, Adams E, Cabello R, Clark JL, Konda K, Perez-Brumer A (2023). PrEP policy implementation gaps and opportunities in Latin America and the Caribbean: a scoping review. Ther Adv Infect Dis.

[19] Baytner-Zamir R, Lorber M, Hermoni D (2014). Assessment of the knowledge and attitudes regarding HIV/AIDS among pre-clinical medical students in Israel. BMC Res Notes.

[20] Sallam M, Alabbadi AM, Abdel-Razeq S, Battah K, Malkawi L, Al-Abbadi MA, Mahafzah A (2022). HIV knowledge and stigmatizing attitude towards people living with HIV/AIDS among medical students in Jordan. Int J Environ Res Public Health.

[21] Ljubas D, Škornjak H, Božičević I (2024). Knowledge, attitudes and beliefs regarding HIV among medical students in Zagreb, Croatia. BMC Med Educ.

[22] Platten M, Pham HN, Nguyen HV, Nguyen NT, Le GM (2014). Knowledge of HIV and factors associated with attitudes towards HIV among final-year medical students at Hanoi Medical University in Vietnam. BMC Public Health.

[23] Bunting SR, Feinstein BA, Hazra A, Sheth NK, Garber SS (2021). Knowledge of HIV and HIV pre-exposure prophylaxis among medical and pharmacy students: a national, multi-site, cross-sectional study. Prev Med Rep.

[24] Przybyla S, Fillo J, Kamper-DeMarco K, Bleasdale J, Parks K, Klasko-Foster L, Morse D (2021). HIV pre-exposure prophylaxis (PrEP) knowledge, familiarity, and attitudes among United States healthcare professional students: a cross-sectional study. Prev Med Rep.

